# Artificial Intelligence Image-Diagnosis for Female Genital Schistosomiasis

**DOI:** 10.1016/j.mcpdig.2025.100245

**Published:** 2025-06-21

**Authors:** Jiayuan Zhu, J. Alison Noble, Mireille Gomes

**Affiliations:** a Department of Engineering Science, University of Oxford, UK; b Department of Global Health & Health Equity, Ares Trading S.A., an affiliate of Merck KGaA, Darmstadt, Germany

## Abstract

**Objective:**

To introduce a novel, artificial Intelligence (AI), deep learning-based application for automated diagnosis of female genital schistosomiasis (FGS), a disease estimated to affect around 56 million women and girls in sub-Saharan Africa.

**Patients and Methods:**

This study focused on cervical images collected from a high endemic FGS area in Cameroon, from August 1, 2020 to August 31, 2021. We applied the You Only Look Once deep learning model and employed a 5-fold cross-validation approach, accompanied by sensitivity analysis, to optimize model performance.

**Results:**

The model achieved a sensitivity of 0.96 (76/78) and an accuracy of 0.78 (97/125), demonstrating improved performance over an existing, non-AI-based, computerized image diagnostic method, which has a sensitivity of 0.94 (73/78) but an accuracy of 0.58 (73/125) on the same dataset. In addition, the AI model significantly reduced processing time, decreasing from 47 minutes to under 90 seconds for testing 250 images.

**Conclusion:**

This study highlights the potential of deep learning-based models for automated diagnosis for FGS while reducing the reliance on specialized clinical expertise. It also underscores the need for further work to address current limitations of such AI-based methods for FGS diagnosis.

According to the World Health Organization, schistosomiasis transmission has been detected in 78 countries and is endemic in 52 countries, with the majority of cases in sub-Saharan Africa.^1^ Globally, ∼230 million people require preventive treatment, with an estimated 700 million population at risk owing to inadequate hygiene and contact with infected water.^1^^,^^2^ As a result, schistosomiasis is the second most widespread parasitic disease after malaria and poses significant public health and economic burdens.^3^

Among its manifestations, female genital schistosomiasis (FGS) is particularly detrimental. Caused by chronic infection with *Schistosoma haematobium*, FGS results from contact with parasite-contaminated freshwater. It affects an estimated 56 million women and girls in sub-Saharan Africa, yet remains a neglected sexual and reproductive health issue.^4^ Symptoms include genital itching, vaginal discharge, and bleeding, with long-term complications, such as sub-fertility, infertility, ectopic pregnancies, spontaneous abortions, and, in rare cases, maternal death.^5^ Lesions may appear on the cervix, vagina, vulva, and in severe cases, extend to the ovaries and fallopian tubes. The FGS also increases susceptibility to sexually transmitted infections, particularly HIV and human papillomavirus, and may influence immune response and disease progression.^6^

Despite its severe health implications, FGS remains underdiagnosed,^5^ primarily due to the lack of widely available and specific diagnostic tools. While conducting biopsies to detect *S haematobium*, eggs are considered the most accurate approach to diagnose FGS yet it is often impractical due to its high cost and high invasive nature.^2^ Instead, the current practical FGS diagnosis relies on visual inspection of lesions on the cervix and vaginal wall.^7^ This approach is further limited by the availability of health care infrastructure and trained personnel in many endemic countries. To address these challenges, there is a pressing need for more accessible and accurate diagnostic tools.^8^ Computerized image-based diagnostic systems offer a promising solution, as they can be deployed in resource-limited settings with minimal infrastructure, for example, through smartphones. To the best of our knowledge, only 2 published studies,^9^^,^^10^ both from the same research group, have explored computerized image analysis tools for FGS. These studies developed adaptive thresholding and morphological analysis techniques on cervical and vaginal images, laying foundational work for advancing automated diagnostic tools. However, neither study incorporated deep learning, indicating potential to explore AI-based diagnostic approach for FGS, particularly in resource-limited settings.

In contrast, considerable advancements have been made in computerized image analysis for cervical cancer, which shares common screening methodologies with FGS. The 2023 literature review by Jin et al^2^ suggests that the progress in computerized image analysis for cervical cancer could be leveraged to improve FGS diagnosis. Recently, more advanced AI-based, deep learning techniques have pushed the boundaries of cervical cancer diagnosis further. For example, You Only Look Once (YOLO) architectures have shown strong performance in real-time detection and classification of abnormalities in cervical screening images.^11^^,^^12^

Building on the progress, this paper presents what is, to the best of our knowledge, the first application of a deep learning method to automated FGS using cervical images. We apply the YOLO architecture, with demonstrated success for cervical cancer diagnosis, to FGS.

## Patients and Methods

### Data Collection

The cervical images used to investigate FGS characteristics were collected by the CIRES (Centre International de Recherches, d’Enseignements et de Soins—local association) clinical team in Cameroon from August 2020, to August 2021, involving 629 participants. We accessed the data after the original study^13^ was completed to develop an AI-based application for FGS diagnosis, which was not among the original study objectives. That study focused on evaluating the feasibility and acceptability of integrating FGS screening and treatment into HIV care; comparing the effectiveness of systematic FGS screening through an HIV clinic versus selective FGS screening by a gynecological mobile service; and assessing the potential for remote FGS diagnostic support by telemedicine. More details are provided in Supplemental Appendix (available online at https://www.mcpdigitalhealth.org/).

**Figure 1:**
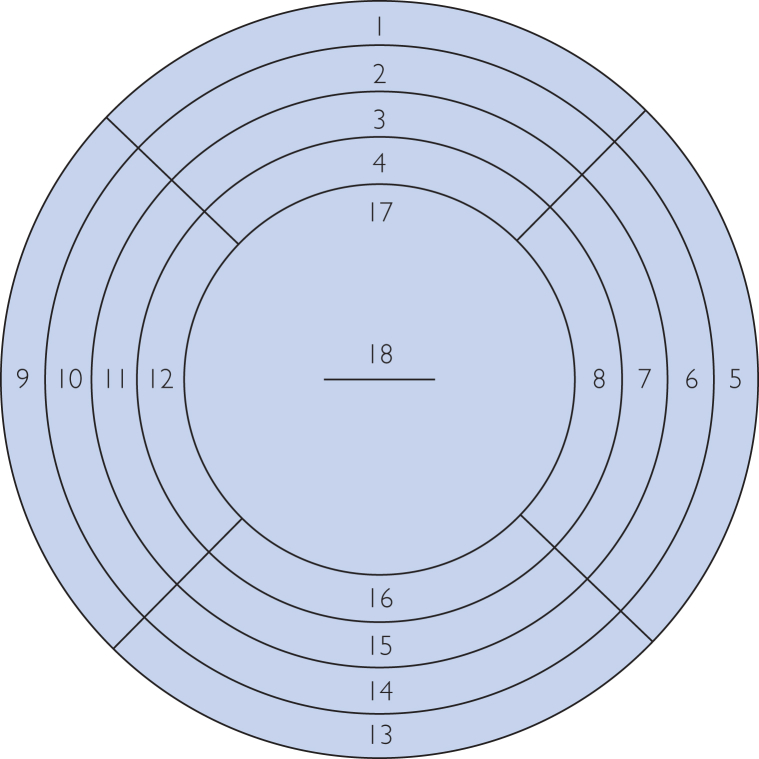
Labeled diagram of 18 anatomical regions used by FGS examiners to identify pathological lesion locations on the cervix. Region 18 represents the cervical center. FGS, female genital schistosomiasis.

**Figure 2:**
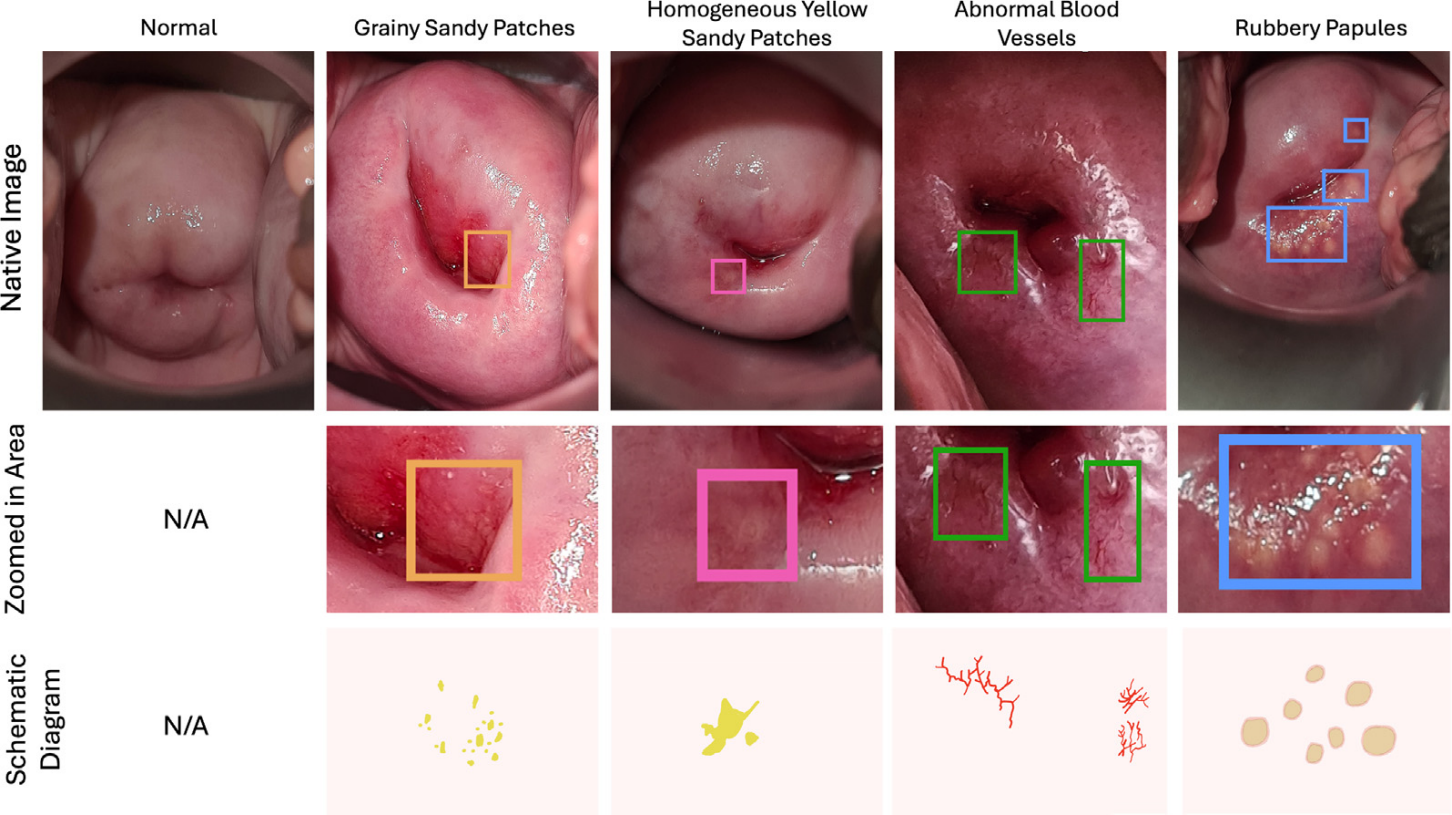
Images highlighting symptom areas related to FGS. The first row displays the full captured views, with bounding boxes marking specific symptom regions. The second row provides zoomed-in views of these highlighted areas. The third row presents schematic diagrams illustrating characteristic FGS symptoms, corresponding to the regions identified in the zoomed-in images. FGS, female genital schistosomiasis.

As part of the original study, an FGS expert examiner and a non-expert examiner independently assessed whether each participant was FGS-positive or negative. Both examiners labeled a diagram with 18 anatomical cervical regions (Figure 1), identifying pathological lesion locations. As shown in Figure 2, cervical lesions in FGS can be categorized into 4 types: grainy sandy patches, homogeneous yellow sandy patches, abnormal blood vessels, and rubbery papules.^5^ The presence of any of these features supports FGS diagnosis.

The non-FGS expert examiner, who was inexperienced in diagnosing FGS but was trained for 8 hours by the expert examiner, performed on-site visual inspections. This examiner captured 2 unstained cervical images per participant using a Samsung Galaxy S20. The smartphone’s high-resolution camera, with built-in magnification and light optimization features, was considered sufficient based on previous studies.^14^ The expert examiner diagnosed FGS based solely on these 2 images sent from the non-FGS expert examiner. To ensure independent diagnosis, each examiner made their assessment without accessing the other’s labeled diagram annotations or diagnosis.

Thus, for each participant, the dataset contains 2 cervical images, along with symptom types, locations of each symptom on the 18 anatomical regions (Figure 1), and FGS-positive/negative diagnosis from each examiner. The dataset does not identify lesions caused by other conditions like sexually transmitted infections or cervical cancer, which may confound the FGS diagnosis by both examiners and computational models. We recognize that this is a limitation of this dataset.

### Examiners’ Data Analysis

Of the total 629 cases, the non-FGS expert examiner identified 341 positive FGS cases, whereas the expert examiner diagnosed 367. They agreed in 571 cases (90.8%), including 325 positive cases and 246 negative cases. As shown in Table 1, both examiners showed variability in identifying symptom types, possibly due to image artifacts (eg lighting), or differences between direct visual inspection and interpretation of the 2D images, etc.

**Table 1:** Number of Individuals Diagnosed as FGS-Positive, Categorized by Symptom Type and Identified by 2 Examiners^a^

Examiner expertise	Grainy sandy patches	Homogeneous yellow sandy patches	Abnormal blood vessels	Rubbery papules	Overall
Non-FGS expert examiner	134 (39.3%)	124 (36.4%)	264 (77.4%)	77 (22.6%)	341
Expert examiner	265 (72.2%)	155 (42.2%)	294 (80.1%)	79 (21.5%)	367
Agreement	124 (92.5%)	94 (75.8%)	253 (95.8%)	55 (71.4%)	325 (95.3%)

a The table shows the total number of cases for each symptom type, the percentage relative to the overall FGS-positive diagnoses, and the agreement between the examiners.

### Data Preprocessing

As the expert examiner was more experienced and made diagnoses solely based on images, we used their annotations as the reference. Specifically, the expert examiner recorded lesion types and locations using an 18-region cervical diagram (Figure 1). On the basis of this, we manually annotated 734 native cervix images from 367 FGS-positive participants (2 images each) using the Computer Vision Annotation Tool platform.^15^ These positive cases were diagnosed by the expert examiner, and no symptoms were annotated on the images where the expert examiner identified them as FGS negative. Given the complex and variable shapes of the FGS symptoms, precise pixel-level annotation was challenging. As such, we opted to use bounding boxes to denote the presence of symptoms (Figure 2).

The manual annotations included 584 bounding boxes for grainy sandy patches, 426 for homogeneous yellow sandy patches, 1112 for abnormal blood vessels, and 236 for rubbery papules. Although a similar number of participants were diagnosed with grainy sandy patches (265) and abnormal blood vessels (294), the count of characteristics annotated per image varied considerably. This discrepancy is primarily due to the frequent detection of multiple abnormal blood vessels in a single image.

We did face challenges annotating the images, which subsequently may impact the computerized diagnostic tool’s performance. For example, specular reflection complicates the identification of grainy sandy patches, as it often appears similar to the scattered yellow dots. In addition, the labeling numbers did not always align perfectly with the actual visual representations, suggesting a possible shift or broadening of the symptom’s definition. Furthermore, misclassification may have occurred due to similarities between symptoms, such as grainy sandy patches being mistaken for homogeneous yellow sandy patches or rubbery papules. In about 10% of cases, we struggled to identify the specific symptoms in the examiner-labeled regions, especially in grainy and homogeneous sandy patches. Some abnormal blood vessels were missed due to bounding box limitations, though their distinct visual characteristics simplified their identification compared to other features.

### Model Architecture

In this study, we apply the YOLO architecture^16^ to develop a deep learning-based image diagnostic tool for FGS, building on its success in cervical cancer detection. The YOLO is a state-of-the-art real-time object detection method that processes entire images in one pass, simultaneously predicting bounding boxes and symptom probabilities. By capturing local features within the bounding box and global characteristics across the entire image, YOLO offers high efficiency and speed, making it well-suited for FGS diagnosis in resource-limited settings.

### Evaluation Matrix

To evaluate the performance of our YOLO-based diagnostic tool for FGS, we consider key metrics: accuracy, sensitivity, specificity, precision, and F1 score. TP, TN, FP, and FN represent true/false positives and negatives. Accuracy reflects overall correct predictions, sensitivity evaluates the ability to correctly detect FGS-positive cases, and specificity indicates correct identification of non-FGS cases. Precision is the proportion of true positives among all positive predictions, whereas the F1 score is the harmonic mean of precision and sensitivity, providing a balanced performance metric. In the FGS diagnosis context, we prioritize sensitivity over specificity to minimize missed FGS-positive cases, which is crucial for clinical follow-up.^2^

$$\text{Accuracy}=\frac{TP+TN}{TP+TN+FP+FN}$$

$$\text{Sensitivity}=\frac{T}{TP+FN}$$

$$\text{Specificity}=\frac{TN}{TN+FP}$$

$$\text{Precision}=\frac{TP}{TP+FP}$$

$$F_{1}=2\times \frac{\text{Precision}\times \text{Sensitivity}}{\text{Precision}+\text{Sensitivity}}$$

### Training and Testing Process

To develop our YOLO-based diagnostic tool for FGS, we divided our dataset of 629 individuals into training and testing sets, allocating 125 individuals (∼20%) for testing and the remaining data for training. Among the 250 testing images, 56 (22.4%), 31 (12.4%), 62 (24.8%), and 17 (6.8%) images contain grainy sandy patches, homogeneous sandy patches, abnormal vessels, and rubbery papules, respectively, based on the expert examiner’s review. These proportions are similar to the 504 individuals in the training set, where of the FGS-positive cases, 208 (20.7%), 123 (12.2%), 231 (23.0%), and 61 (6.1%) images exhibit these symptoms.

During the training phase, we employed a 5-fold cross-validation to select optimal hyperparameters. In this method, the training dataset is randomly divided into 5 subsets, with each subset serving as the validation set once and the remaining 4 subsets for training. This approach allows robust hyperparameter performance estimation and reduces overfitting by exposing the model to diverse data splits, thereby enhancing its ability to perform well on unseen testing data.

After selecting the best hyperparameters, we trained the final model on the entire training dataset and evaluated it on the testing data. Manually annotated bounding boxes were used during training, whereas the model automatically predicted bounding boxes for the testing set. This process supports the development of a reliable and accurate diagnostic tool capable of identifying FGS lesions with minimized bias and improved generalizability.

### Hyperparameter Tuning

In our implementation of the YOLO architecture for FGS diagnosis, we fine-tuned the confidence parameter to optimize detection performance. This parameter combines box confidence (probability that a model-predicted bounding box contains an object) and symptom confidence (probability that the model detected object belongs to a specific symptom type), reflecting both detection and classification ability.

Using 5-fold validation on the training data, we conducted a sensitivity analysis by varying the confidence parameter from 0.001 to 0.2 in 0.01 increments. A confidence parameter of 0.02 yielded the best average F1 score of 0.82 (standard deviation=0.02) and achieved average sensitivity of 0.91 (standard deviation=0.05) across the 5-folds. We selected this lower confidence threshold to prioritize detecting true positive, acknowledging the trade-off of potentially higher false positives. As previously discussed, sensitivity is prioritized over specificity in this context. Hyperparameters can be adjusted depending on the specific context and objectives of the application (details in Supplemental Appendix).

## Results

### FGS Diagnosis Result—Symptom Type

Table 2 presents the performance of the YOLO-based FGS diagnostic tool across different symptom types. The aggregated performance row reflects cases where the presence of any symptom is considered positive. This evaluation was conducted across 250 images from 125 individuals (2 images each) in the testing set. The aggregated model performance achieved an accuracy of 0.78 (196/250), sensitivity of 0.92 (143/156), specificity of 0.56 (53/94), precision of 0.78 (143/184), and an F1 score of 0.84. These results indicate that the model is overall good in detecting FGS symptoms, particularly excelling in sensitivity, which is crucial for identifying subtle symptoms. However, the variability in performance across different symptom types and the lower specificity underscore the need for further refinement to enhance diagnostic accuracy and consistency across all symptom categories.

**Table 2:** Performance Metrics of YOLO-Based Diagnostic Approach For Different Types of FGS Symptoms^a^^,^^b^

Symptom type	Accuracy	Sensitivity	Specificity	Precision	F1 score
Grainy sandy patches	0.60 (151/250)	0.67 (72/107)	0.55 (79/143)	0.53 (72/136)	0.60
Homogeneous yellow sandy patches	0.72 (180/250)	0.60 (39/65)	0.76 (141/185)	0.47 (39/83)	0.53
Abnormal blood vessels	0.78 (195/250)	0.83 (102/123)	0.73 (93/128)	0.75 (102/136)	0.79
Rubbery papules	0.84 (209/250)	0.56 (19/34)	0.88 (190/216)	0.42 (19/45)	0.48
Aggregated performance	0.78 (196/250)	0.92 (143/156)	0.56 (53/94)	0.78 (143/184)	0.84

a The table presents Accuracy, Sensitivity, Specificity, Precision, and F1 Score for various symptom types, including grainy sandy patches, homogeneous yellow sandy patches, abnormal blood vessels, and rubbery papules, evaluated across 250 images from 125 individuals. The aggregated performance row summarizes the model’s overall performance across all symptom types, considering the presence of at least one symptom as FGS-positive.

b Abbreviation: YOLO, you only look once.

### FGS Diagnosis Result—Individual Patient

To evaluate the YOLO-based diagnostic tool at the individual level, we combined results from each participant’s 2 images. An individual was considered FGS-positive if at least one image showed detected symptoms. Our model achieved an overall accuracy of 0.78 (97/125) and a sensitivity of 0.96 (76/78). However, the specificity of 0.47 (22/47) indicates some limitations in correctly identifying nonaffected cases. The F1 score of 0.84 underscores the model’s strong balance between precision and sensitivity and ability to distinguish between affected and nonaffected cases.

### Bounding Box Area

We analyzed the model-predicted bounding boxes on the testing set by examining both the number and area of bounding boxes per image, grouped by examiner agreement. When both examiners agreed on a positive FGS diagnosis, the model-predicted an average of 13.62 bounding boxes per image (standard deviation=13.14), suggesting strong and consistent symptom detection. In cases of disagreement, the average number of predicted bounding boxes dropped to 9.06 (standard deviation=5.80) per image, possibly reflecting uncertainties or less pronounced symptoms. When both examiners agreed on a negative diagnosis, the average number of model-predicted bounding boxes dropped significantly to 1.88 (standard deviation=3.39) per image, indicating the model’s ability to correctly identify nonaffected cases and reinforce its specificity.

The analysis of model-predicted bounding box areas further supports this. For positive consensus cases, boxes covered 0.84% of the total image area on average, compared with 0.64% of the total image area in disagreement cases and just 0.44% of the total image area in negative consensus cases. These trends suggest the model detects more and larger regions when symptoms are more evident and agreed on, while predicting fewer and smaller regions in uncertain or negative cases. Overall, this analysis highlights the model’s ability to reflect varying levels of symptom presence, with performance aligned to examiner consensus. The understanding of bounding box metrics may help in refining the diagnostic tool and improving its accuracy and reliability in identifying FGS symptoms.

### Comparison with Color Analysis-based FGS Model

We compared our YOLO-based deep learning model with the only previously published computerized image analysis tool for FGS, which is based on color analysis without deep learning.^10^ That method employs adaptive thresholding, setting a reference from the mean mucosal color to adjust for varying exposure and color balance, while also masking reflections within a defined region of interest.

For comparison, we implemented this color analysis approach by downloading the model from the first author, (Sigve Holmen’s GitHub repository, https://github.com/sigveholmen/cervigram-analyses) and followed the instructions outlined. This repository contains plugins and macros for ImageJ (https://imagej.nih.gov/ij/), which we used to execute the analyses and obtain visualized results for comparison. The color analysis method achieved an accuracy of 0.58 (73/125), which is significantly lower than our YOLO-based model’s 0.78 (97/125). Although the color analysis method had a slightly lower sensitivity at 0.94 (73/78) compared with 0.96 (76/78), it failed to identify any true negatives, resulting in a specificity of 0 as compared with 0.47 (22/47) of the YOLO-based models. This suggests that the previous nondeep learning-based approach may overpredict FGS cases, struggling to correctly identify nonaffected individuals. The precision of the color analysis method was 0.61 (73/120), compared with 0.75 (75/100) for the YOLO-based model, highlighting an enhanced ability of the YOLO-based tool to detect true positive cases. The F1 score for the color analysis-based method was 0.74, considerably lower than the 0.84 achieved by the YOLO-based approach. Overall, the color analysis method exhibited important limitations in specificity and precision, leading to an overestimation of positive cases. In contrast, the YOLO-based tool provides a relatively balanced and reliable diagnostic performance, reducing processing time from 47 minutes to under 90 seconds for testing 250 images, while maintaining high accuracy and sensitivity.

It is important to note that the color analysis-based method primarily detects sandy patches and does not capture abnormal blood vessels or rubbery papules, limiting its diagnostic capability and resulting in an incomplete comparison between the models.

**Figure 3:**
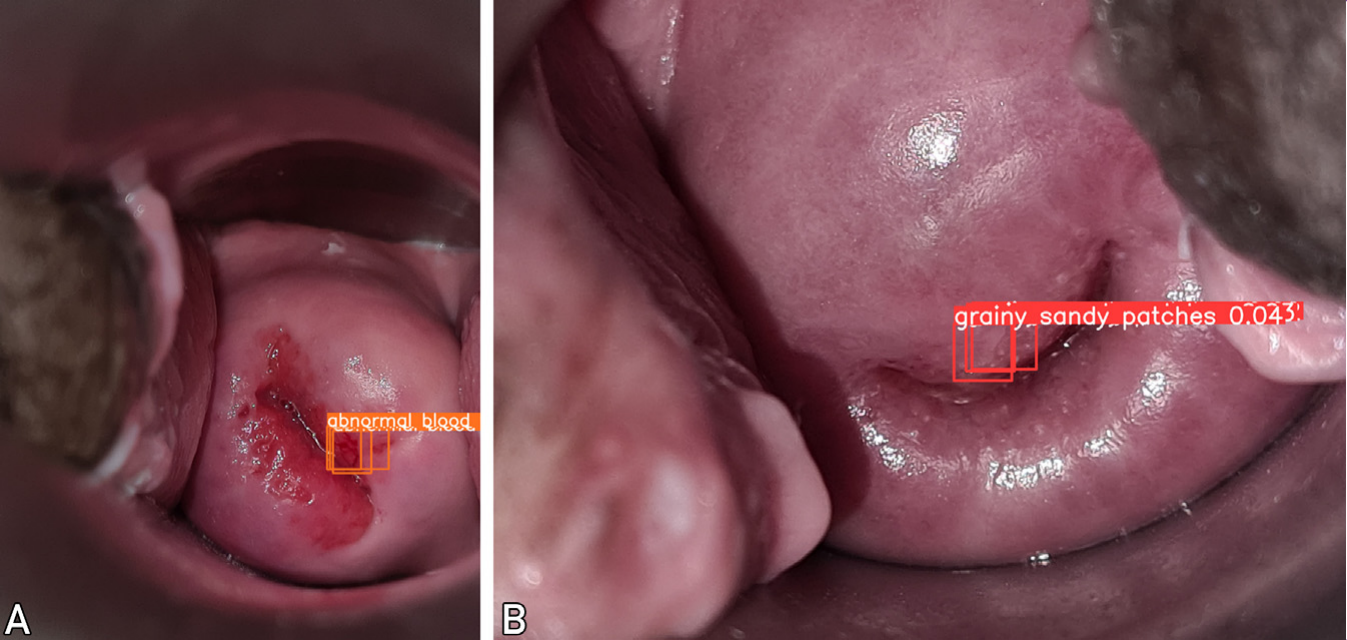
(A) A cervix image detected as FGS-positive from our method with abnormal vessels symptom. (B) A cervix image detected as FGS-positive from our method with grainy sandy patches symptom. FGS, female genital schistosomiasis.

Figure 3A and B illustrate 2 visualizations of FGS predictions generated by the YOLO-based approach. It shows precise localization of symptomatic regions within the central cervix, aligning closely with clinical expectations. However, the color analysis approach has the tendency to capture irrelevant areas beyond the cervix, including operational instruments or cervical walls. The YOLO-based model’s enhanced focus on relevant anatomical structures supports more accurate and interpretable FGS diagnosis, improving practical applicability and diagnostic confidence in real-world clinical settings.

## Conclusion

In this study, we introduced what is, to our knowledge, the first AI-based deep learning application for diagnosing FGS from cervical images. Our approach considerably improves diagnostic accuracy, precision, and processing time compared with previous nondeep learning computerized FGS diagnostic methods.^6^ With a sensitivity of 0.96 (76/78), the model effectively minimizes false negatives, which is crucial for ensuring FGS-positives are not missed while maintaining a reasonable diagnostic balance with a specificity of 0.47 (22/47). We employed 5-fold cross-validation and sensitivity analysis to enhance model robustness and reliability.

However, several limitations remain. It is unclear how well the model generalizes to datasets captured in different regions or with different imaging devices. The current model was developed based on a single examiner’s diagnosis, limiting inter-observer variability assessment. The relatively small dataset and use of only 2 static images per participant, unlike the dynamic multi-angle views available in live colposcopic examinations, also constrain model generalizability. In addition, the examiners may have been hypersensitive toward looking for FGS-specific changes on the cervix, introducing inherent biases. These factors highlight the need for future validation on larger and more diverse datasets to better evaluate the model’s robustness and generalizability.

Further challenges include bounding box overprediction, model interpretability, and the practical deployment in resource-limited settings. Overprediction could be mitigated by integrating segmentation techniques to better capture symptom shape and size. Improving interpretability is essential for clinical adoption, as clinicians need clear explanations of the model’s decisions. For deployment in resource-limited areas, the development of efficient, AI-based diagnostic tools that can operate with minimal computational and electronic power is critical, underscoring the importance of collaboration with local health care providers. Although this study focused on detecting FGS-specific features, future research could expand the diagnostic scope to distinguish FGS from other genital conditions, such as inflammation, HPV, squamous intraepithelial lesions, and cervical cancer lesions.

In conclusion, our YOLO-based FGS diagnostic tool represents an important step forward in the computerized detection of FGS. Future work should focus on addressing the identified challenges to further enhance AI-based diagnostic performance and applicability, ultimately contributing to better health care outcomes for those affected by FGS.

## Potential Competing Interests

Dr Zhu is funded by Engineering and Physical Sciences Research Council (EPSRC) and the healthcare business of Merck KGaA, Darmstadt, Germany. Dr Gomes worked for Ares Trading S.A., Eysins, Switzerland, an affiliate of Merck KGaA, Darmstadt, Germany. Dr Noble reports no competing interests.
