# Supplementary material for: Artificial Intelligence Image-Diagnosis for Female Genital Schistosomiasis
Source: Mayo Clin Proc Digit Health. 2025 Jun 21;3(3):100245. doi: 10.1016/j.mcpdig.2025.100245 (PMC12381638; doi:10.1016/j.mcpdig.2025.100245)
Supplement: Supplementary Data [file mmc1.pdf]

Supplementary

1 Data Collection

The data collection was conducted in Bertoua and Doume Health Districts in the East Region of Cameroon. A total of 1242 women were offered FGS screening in two different settings. In the first setting, 618 women were approached through community health workers, who informed them about the opportunity for FGS screening in a gynecological mobile clinic that visited three communities: Dimako Nord, Dimako Sud, and Mandjou. These villages were targeted due to the higher prevalence of *S. Haematobium* identified in a previous micromapping study. 378 women from the gynecological mobile clinic enrolled in the study. The second setting consisted of HIV static clinics located in a main hospital or secondary health centers dedicated to the follow-up of HIV-positive women. A total of 624 women were offered participation in these static HIV clinics, which are located in the Bertoua and Doume districts. Among them, 251 women from the HIV clinics were enrolled in the study. In both settings, the sampling method was convenient. Women were excluded if they were outside the target age range, pregnant, or did not consent to participate in the study. The main reason for non-enrollment was refusal to participate in the screening and the study. All enrolled participants underwent screening for FGS lesions, with 56% (212 women) testing positive in the gynecological mobile clinic and 51% (129 women) in the HIV clinics.

2 Hyperparameter Tuning

The results illustrate the trade-offs between sensitivity (Fig 1), specificity (Fig 2), and F1 score (Fig 3) as the confidence threshold parameter is adjusted. At very low thresholds (e.g., 0.001), the model achieves high sensitivity (up to 1.00), which maximises the detection of true positives but compromises specificity, indicating a higher likelihood of false positives. As the threshold increases, specificity improves significantly, reaching 0.98 at a threshold of 0.19, demonstrating fewer false positives. However, this comes at the expense of sensitivity, which drops to 0.23. The F1 score, which balances precision and recall, peaks around a threshold of 0.02 with a value of 0.82 and gradually declines as the threshold increases.

If the objective is to achieve a balanced sensitivity and specificity, a higher threshold like 0.04 would be suitable. At this threshold, the model achieves a balanced sensitivity of 0.76 and an F1 score of 0.78 while maintaining a reasonable specificity of 0.76. This threshold represents a compromise that still captures most true positives without overly

sacrificing specificity.

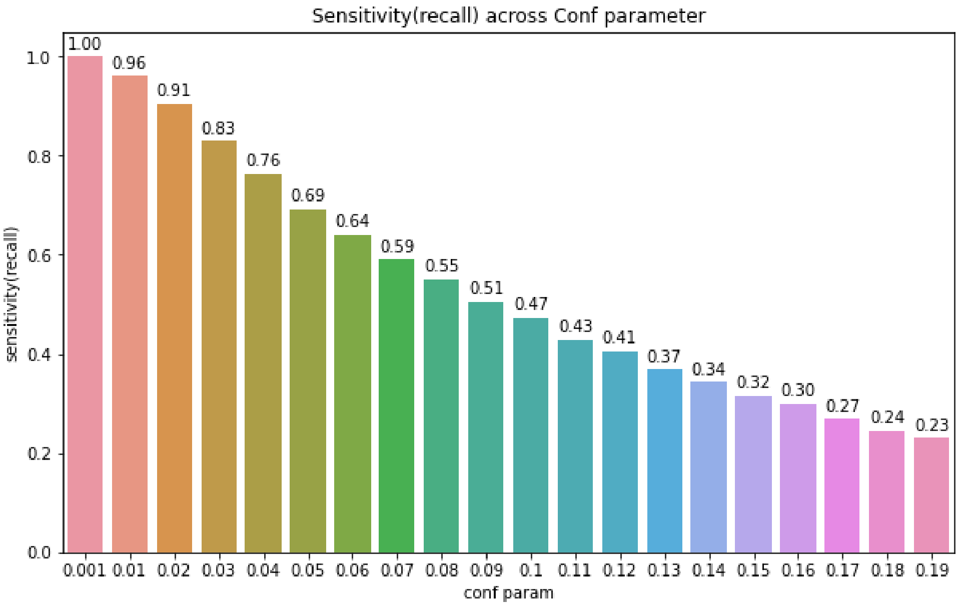

Figure 1: Sensitivity analysis measured by sensitivity score.

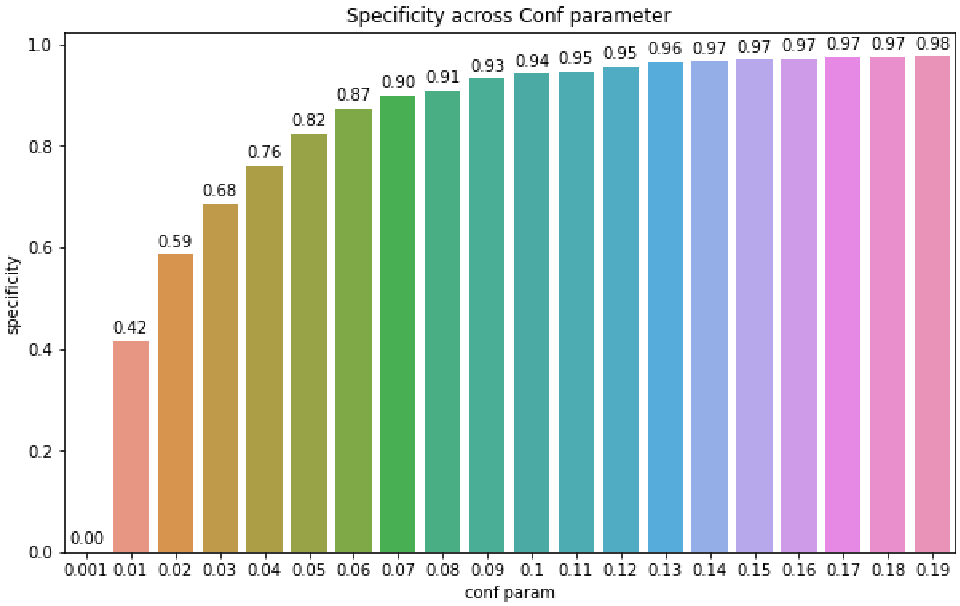

Figure 2: Sensitivity analysis measured by specificity score.

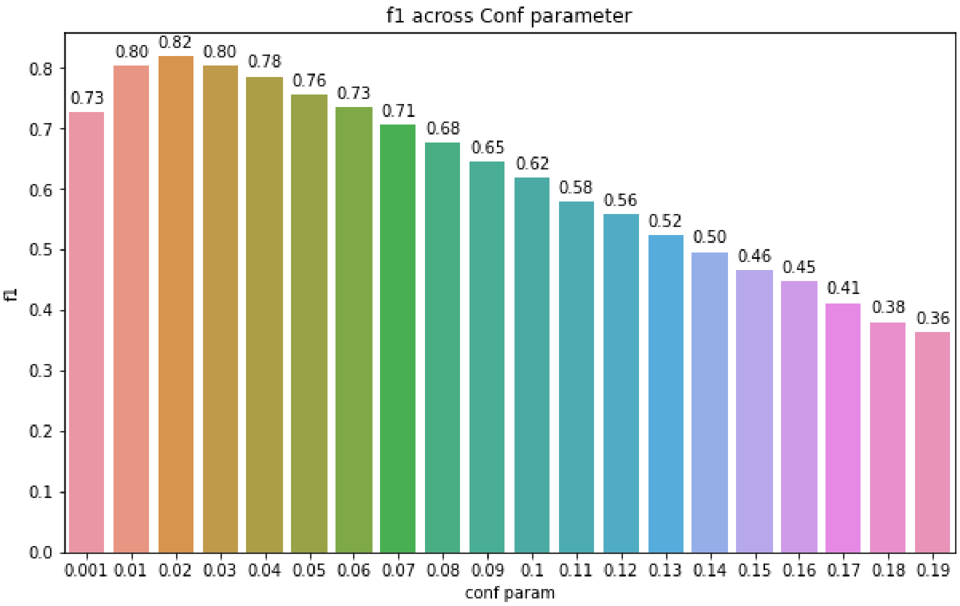

Figure 3: Sensitivity analysis measured by F1 score.
